# Engagement with health in the climate reports of top corporations

**DOI:** 10.1016/j.joclim.2025.100643

**Published:** 2026-01-07

**Authors:** Barbara P F Davis, Paul J Beggs, Petra L Graham

**Affiliations:** a School of Natural Sciences, Faculty of Science and Engineering, Macquarie University, Sydney, New South Wales 2109, Australia; b School of Mathematical and Physical Sciences, Faculty of Science and Engineering, Macquarie University, Sydney, New South Wales 2109, Australia

**Keywords:** Climate change, Health, Impact, Adaptation, Mitigation, Australia

## Abstract

**Introduction:**

Climate change threatens health and wellbeing globally, requiring the combined attention of multiple societal actors. To assess corporate participation, this study’s objective was to quantify engagement with health in publicly available corporate climate reports in Australia.

**Methods:**

Climate reports from Australia’s top 500 corporations as determined in September 2023 were obtained in September 2023-April 2024 from their websites or replies to email requests. Content analysis was used to locate and quantify health and other words within the reports. The characteristics of corporations with climate reports were compared to those without climate reports in terms of market capitalisation, sector, emissions reduction, and country of incorporation.

**Results:**

Among the top 500 corporations, 51 (10.2 %) had standalone climate reports available by April 2024. Climate reports were predominately found for larger corporations and those with the highest emissions per million AUD market capitalisation. The sector with the greatest proportion of climate reports was the utilities sector (62.5 %). Companies without and with climate reports had a non-significant difference in median absolute change in tonnes of CO_2_ equivalents emissions per million AUD market capitalisation (-0.2 vs -6.9, *p* = 0.706) from 2017–2018 to 2022–2023. Fifty (96.2 %) climate reports mentioned a climate change-related health word. Two-thirds (*n* = 34) of climate reports referred to specific actions to tackle health impacts.

**Conclusion:**

While a relatively small percentage of top Australian companies, representing a range of sectors, had standalone climate reports in 2023–2024, health impacts and adaptation to reduce these impacts are acknowledged and discussed in most of these climate reports.

## Highlights

• Health content in climate reports from top 500 companies in Australia was assessed.
• 51/500 companies (10.2 %) had climate reports in April 2024.
• Climate reports tended to be produced by larger companies.
• Almost all climate reports included health content.
• Two-thirds of the reports referred to specific actions to tackle health impacts.

## Graphical abstract

**Figure:**
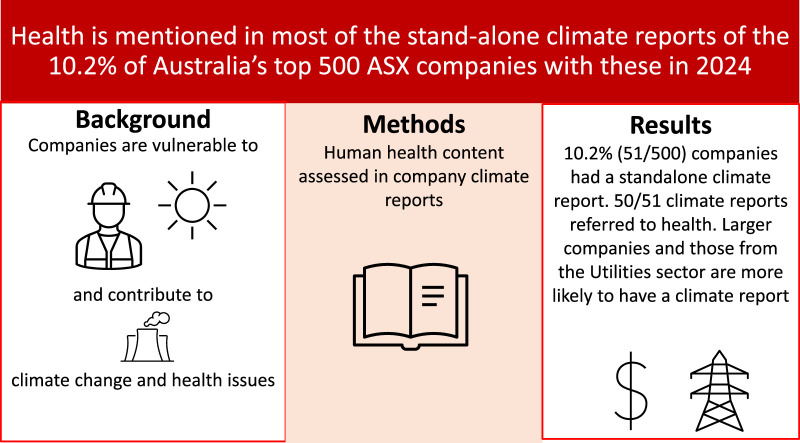

## 1 Introduction

In 2015, 195 countries signed the Paris Agreement [1] thereby agreeing to aim to achieve net zero emissions by 2050 (Article 4.1) and limit the global average temperature increase to 1.5 °C above pre-industrial levels (Article 2.1.a). Despite this almost unanimous agreement between nations, temperatures are projected to rise by 2.5 °C by 2100 [2]. It is well recognised that global warming poses a direct existential threat to humans [3].

Observed and projected climate change-related health problems may be multiple, simultaneous and compounding especially for vulnerable people [4] such as rural low-income communities; people displaced by conflict; people living in informal settlements; indigenous people; the elderly; and people with pre-existing chronic diseases. Climate change-related health risks include heat stress from outdoor activities; drought-related food insecurity and high airborne particulate matter; heat and precipitation-related water contamination and infectious diseases such as dengue and malaria; and health risks from deteriorating socioeconomic conditions due to extreme weather events [5].

Corporations are major contributors to climate change through their emissions, and through their influence on government policy through lobbying and on consumer behaviour through advertising [3,[6], [7], [8]]. With profits outstripping the Gross Domestic Product (GDP) of some small countries [9], corporations potentially have the capacity to contribute to adaptation and mitigation of the health impacts of climate change. Corporations, whose stakeholders consist of employees, shareholders, consumers and communities, are also vulnerable to climate change and by extension its health consequences [10]. However, businesses previously have been criticised for lack of engagement with climate change overall including adaptation to climate change [11] and specifically a lack of engagement with health and climate change [12].

The classification of corporate risks as physical risk, regulatory risk, market risk and reputational risk, as described by Nyberg [13], could apply to climate change-related health risks as follows. For example, the physical risk of climate change is one to which outdoor workers in particular are susceptible [14]. Regulatory risk in relation to climate change is an evolving issue for corporations, particularly in Australia, which has been identified as a “hotspot for climate litigation” [15]. Market risk for corporations could occur if extreme weather events affect the distribution of health products and services [16]. Corporations may be vulnerable to reputational risk if they do not engage with the climate change-related health problems in the communities in which they operate. According to Hiswåls et al. [17], in regards to health alone “there has been increased interest in the role played by business corporate social responsibility (CSR) strategies in promoting the health and wellbeing of internal and external stakeholders”.

In Australia, mandated disclosure by corporations of risk is a legal requirement in the Corporation Act 2001 [18] and Australia has been progressively introducing climate-related disclosures since 2024 [19,20]. Corporations may use as a template climate risk disclosure guidelines such as the Taskforce on Climate Related Financial Disclosures (TCFD) which includes only two references to health (once as a climate-related physical risk and another as a climate-related opportunity) [21]. It was already a requirement in 2023 that high emitting entities/corporations report their emissions to the Australian Clean Energy Regulator according to the National Greenhouse Emission Reporting Act [22].

Companies produce a variety of reports including compulsory annual reports (describing financial governance and sometimes, environmental disclosure [23]), voluntary sustainability reports (covering environmental and social issues [24]), and climate reports. Increasingly promoted are so-called “integrated” reports combining financial and nonfinancial reports [25] thus taking into account financial, social, intellectual and manufactured, human, and natural capital [26]. Climate reports were chosen for this study because they are studied by the Carbon Disclosure Project [27], the Paris Agreement and the Lancet Countdown series of reports on health and climate change [3,5,[28], [29], [30], [31], [32]].

This study aimed, as a proxy for engagement, to 1. assess the heath content of climate reports and 2. categorise corporate engagement with climate change-related health issues as risk assessment, mitigation, and/or adaptation.

## 2 Methods

### 2.1 Data and data collection techniques

This study used the top 500 Australian Securities Exchange (ASX) commercial corporate organisations (companies), by market capitalisation (the value of securities available to shareholders) as determined by those trading on 19th September 2023. Company characteristics were extracted including Global Industry Classification (GIC) Industry Group sector, and country of incorporation. If the sector was not available from the ASX website, it was obtained from the Market Index website [33]. The country of incorporation for the companies was determined from the database DatAnalysis Premium [34] and the ASX foreign entity data website [35].

Scope 1 emissions for the financial years 2017–2018 and 2022–2023 for each of the companies was obtained, where available, from the Australian Federal Government’s greenhouse gas reporting site [36].

For these top 500 companies, publicly available standalone climate reports were collected in the 7 months from 26 September 2023 until 3 April 2024, by searching their websites. If a search function was available on the website, the search term “climate change” was entered. A search function was present in about 200/500 websites. If no climate report was found by this technique, the website’s “governance” section and then the “investor” section was inspected for reports and companies were emailed. Climate reports were defined as reports that focused on climate change. Almost all included the word "climate" in the title, e.g., "Climate Report", "Climate Change", "Climate Action Plan", "Climate Transition Action Plan" or similar. Carbon disclosure project (CDP), TCFD and climate “strategies” documents were also included as climate reports. Only one report was included per company which was the most up to date report regardless of type. Climate policies were not included as a climate report as they tended to be statements of principle. Climate-related sections of other reports such as annual reports or sustainability reports were not included.

The climate report PDFs were converted to plain text using R version 4.3.2 statistical software [37] with the tabuliser package [38]. The quanteda package [39] was then used to process the text in each report by removing punctuation and numbers and converting the text to lowercase to enable a word search.

A dictionary of health words was compiled (Appendix Table A1), based on that used by the Lancet Countdown on Health and Climate Change [3] with the addition of several relevant Australia-specific health words (such as mosquito-borne diseases that are endemic to Australia). Base words were identified such as injur* which would capture all forms, including the singular “injury” and plural form “injuries”.

The quanteda package was then used to determine the total number of words in each report together with the frequency of each health word and all health words. In addition, the frequency of each word in the report (excluding stop words such as articles, pronouns and prepositions) was obtained and a word cloud generated to reveal the overall report focus. Finally, common words and categories of words in the reports were noted from which the main priorities, themes and motivators could be determined.

References to health were also categorised based on whether their broader context referred to climate change and human health impacts, mitigation, or adaptation. “Mitigation” in this case is referring to the health benefits of reducing emissions. “Adaptation” is used broadly to be any related action to reduce the health impacts currently or in the future and includes disaster relief. Adaptation and mitigation show the company is doing more than acknowledging a problem.

### 2.2 Analysis

Descriptive statistics (median and interquartile range (IQR) or count with percentage, as relevant) were used to summarise the characteristics of corporations with and without climate reports and the health content of climate reports.

Wilcoxon rank sum tests were used to assess whether market capitalisation differed between companies with and without a climate report. Scope 1 emissions were standardised for company size (to adjust for change in company size over time) by dividing by market capitalisation to create emissions in tonnes of CO_2_ equivalent per million Australian dollars (AUD). Wilcoxon rank sum tests were used to determine whether the change in standardised emissions or percentage change in emissions between 2017–2018 and 2022–2023 differed between companies with and without a climate report.

Fisher’s exact test was used to determine whether the proportion of companies with a climate report differed between sectors and country of incorporation. P-values <0.05 were considered significant. R 4.3.2 statistical software was used for all analyses.

## 3 Results

Out of the 500 top ASX companies there were 51 (10.2 %) with climate reports. Sixteen reports (31.4 %) had the words *climate change* in their title, twelve titles (23.5 %) related to climate strategy and/or action, ten titles (19.6 %) related to climate-related disclosure including financial disclosure and 6 (11.8 %) were simply titled climate reports. The remaining 7 (13.7 %) had variable titles. Eight reports (15.7 %) identified as being CDP reports, 29 (56.9 %) identified alignment with the TCFD and there was no apparent alignment for the remaining 14 (27.5 %).

**Fig. 1:**
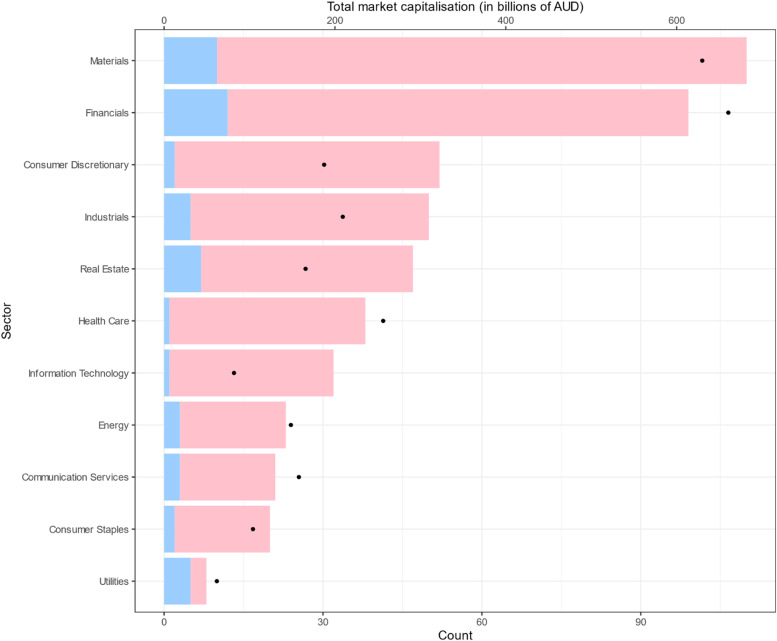
The number of top Australian corporations by sector with (blue) and without (pink) a climate report. The black dots represent total market capitalisation (in billions of AUD) of each sector.

Most of the companies came from the Financials (19.8 %) and Materials sectors (22.0 %) and there was a statistically significant difference in the proportion of companies with a climate report between sectors (*p* = 0.006), with the proportion of companies with reports ranging between 3 % to 63 %. The Utilities sector had the largest proportion of reports 5/8 (63 %) while the Health Care sector had the smallest proportion of reports 1/38 (2.6 %) (Table 1, Fig. 1).

**Table 1:** Characteristics of ASX500 corporations with and without a standalone climate report.

	Overall (*n* = 500)	Climate report absent (*n* = 449)	Climate report present (*n* = 51)	P-value
Market capitalisation 19/9/2023 (AUD billions); median (IQR)	1.07 (0.43, 3.39)	0.89 (0.41, 2.58)	7.35 (2.40, 14.57)	<0.001
GICS sector, n ( %)				0.006
Communication Services	21 (4.2)	18 (85.7)	3 (14.3)	
Consumer Discretionary	52 (10.4)	50 (96.2)	2 (3.8)	
Consumer Staples	20 (4.0)	18 (90.0)	2 (10.0)	
Energy	23 (4.6)	20 (87.0)	3 (13.0)	
Financials	99 (19.8)	87 (87.9)	12 (12.1)	
Health Care	38 (7.6)	37 (97.4)	1 (2.6)	
Industrials	50 (10.0)	45 (90.0)	5 (10.0)	
Information Technology	32 (6.4)	31 (96.9)	1 (3.1)	
Materials	110 (22.0)	100 (90.9)	10 (9.1)	
Real Estate	47 (9.4)	40 (85.1)	7 (14.9)	
Utilities	8 (1.6)	3 (37.5)	5 (62.5)	
Country of incorporation, n ( %)				0.210
Australia	434 (86.8)	390 (89.9)	44 (10.1)	
New Zealand	35 (7.0)	29 (82.9)	6 (17.1)	
USA	11 (2.2)	10 (90.9)	1 (9.1)	
Other	20 (4.0)	20 (100)	0 (0)	
Emissions (Tonnes of CO_2_ equivalents) per million AUD market capitalisation 2017–2018; median (IQR)	67.9 (3.5, 229.5)	43.6 (4.7, 181.9)	77.3 (2.4, 269.4)	0.646
	*n* = 75	*n* = 51	*n* = 24	
Emissions (Tonnes of CO_2_ equivalents) per million AUD market capitalisation 2022–2023; median (IQR)	57.1 (8.8, 191.3)	49.5 (8.2, 153.9)	86.2 (15.6, 223.2)	0.303
	*n* = 81	*n* = 60	*n* = 21	
Change in emissions (tonnes of CO_2_ equivalents) per million AUD market capitalisation from 2017–2018 to 2022–2023; median (IQR)	−0.3 (−46.3, 5.2)	−0.2 (−44.2, 4.0)	−6.9 (−73.2, 8.4)	0.706
	*n* = 65	*n* = 45	*n* = 20	
Percentage change in emissions (tonnes of CO_2_ equivalents) per million AUD market capitalisation from 2017–2018 to 2022–2023; median (IQR)	−20.6 (−43.6, 30.6)	−20.6 (−44.0, 31.8)	−17.1 (−31.4, 15.1)	0.904
	*n* = 65	*n* = 45	*n* = 20

Most companies were incorporated in Australia (86.8 %, Table 1). There was no evidence of a difference in the proportion of companies with a climate report by corporation’s country of incorporation (*p* = 0.210).

Corporations with climate reports had significantly larger median market capitalisation (median 7.35 IQR (2.40, 14.57) [in AUD billions]) than those without reports (median 0.89 IQR (0.41, 2.58) [in AUD billions]), *p* < 0.001, Table 1.

Twenty of the 51 companies with a report (39.2 %) had emissions and market capitalisation data for the years 2017–2018 and 2022–2023 and 45 of the 449 (10.0 %) companies without a report had emissions and market capitalisation data in both years. After adjusting for change in company size over time by standardising by market capitalisation, companies with and without climate reports had a non-significant difference in median absolute (−6.9 vs −0.2 tonnes of CO_2_ equivalent per million AUD market capitalisation, *p* = 0.706, Table 1) and percentage (−17.1 % vs −20.6 %, *p* = 0.904) change in emissions. The reduction in emissions found for these two sets of companies with emissions and market capitalisation data in the two years, differs to the single year emissions results shown in Table 1, which are based on larger sets of companies with emissions and market capitalisation data in just one of the two years.

**Fig. 2:**
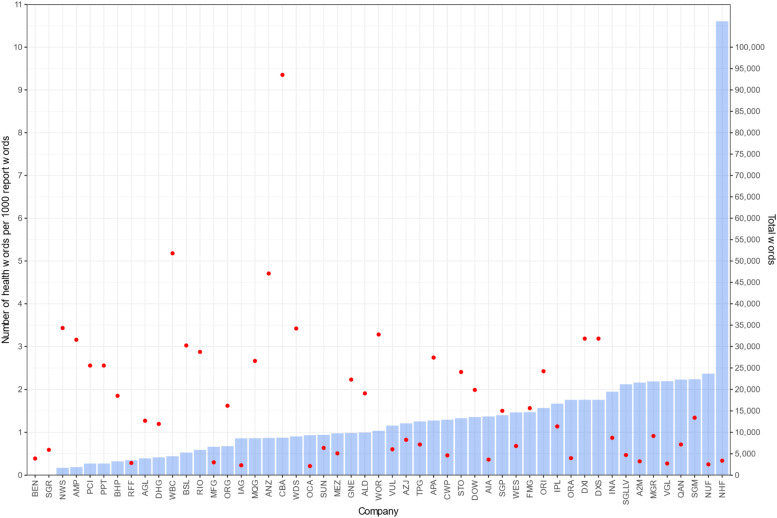
Health words per 1000 total words per climate report (bars) and total number of words per climate report (red dots).

**Fig. 3:**
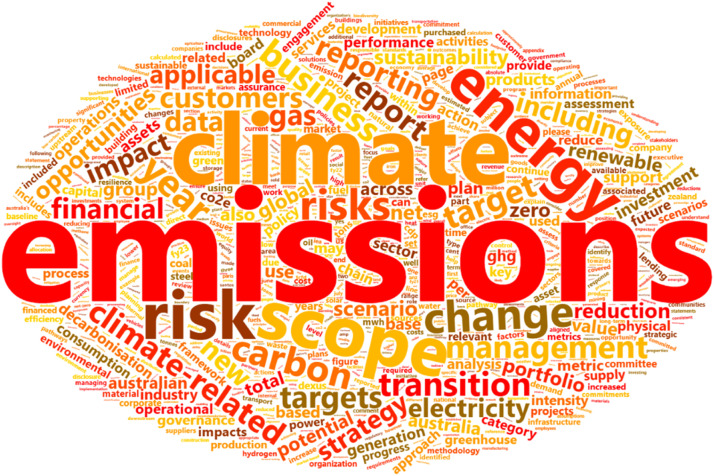
Word cloud showing the top 1000 most frequently used report words (excluding stop words). Larger sized words occurred more frequently.

Reports were variable in length, ranging between 2145 and 93,527 words (Fig. 2, dots). The most common report word was “emissions”, which occurred 11,680 times and report themes largely related to energy and risk (Fig. 3).

A health-related word was mentioned between 1 and 82 times in 49/51 (96.1 %) reports (Appendix Table A1). The rate of health words per 1000 words in the climate reports is shown in Fig. 2 (bars), with one report (from financials company nib group (NHF), a health insurance company) having a much higher rate of health words (10.6 words per 1000 report words) than the other reports (median 1.0 health words per 1000 report words, IQR: 0.6 to 1.6).

The actual word “health” was mentioned from 1 to 26 times in 38/51 reports (75 %). In terms of type of health impacts, the most frequent health words were “safety” which was mentioned from 1 to 22 times in 35/51 reports (69 %), and “depression”, “anxiety” or “stress” which was mentioned from 1 to 32 times in 32/51 reports (63 %). Less frequently mentioned were “heat stroke” or “heat stress” which were mentioned 1 to 10 times in 12/51 reports (24 %), and “air pollution” or “air quality” which were mentioned 1 to 5 times in 9/51 reports (18 %). Many health aspects were rarely or never mentioned including vector-borne and food-borne diseases (Appendix Table A1).

In descending order, the word “emissions” (*n* = 11,680) was ranked 1 as the most frequently used word in the reports, environmental (*n* = 852) was ranked 104; economy (*n* = 425) was ranked 258; heat (*n* = 424) was ranked 260; biodiversity (*n* = 334) was ranked 337 and nature (*n* = 332) was ranked 341. By comparison the most frequent health words were mentioned as follows, safety (*n* = 189) was ranked 627 and health (*n* = 177) was ranked 664.

Overall, 49/51 (96 %) climate reports mentioned internal stakeholders (employees, workers, teams and staff) (100 % if CEO and directors were included) and 49/51 (96 %) climate reports mentioned external stakeholders (customers, communities, clients, consumers, investors and shareholders). Most reports mentioned the TCFD (46/51, 90 %) and just over one third (19/51, 37 %) mentioned the CDP. Words related to legislation occurred in almost all reports (94 %). The Paris Agreement was mentioned in a large majority of reports (43/51, 84 %) (see Appendix Table A1).

Contextual analysis of the reports revealed that many of the corporations are engaging with the 1. impacts of climate change on health, 2. the health benefits of mitigation and 3. adaptation responses, and these are quantified as follows. About two-thirds of the reports mentioned human health impacts (31/51, 60.78 %), and a similar proportion mentioned health adaptation (34/51, 66.67 %). Health mitigation was mentioned in almost half (23/51, 45.10 %). However, none of the reports included all three aspects.

Regarding adaptation, common ways of addressing these impacts or risks were through monitoring, e.g., “heat stress monitoring at our major operating facilities when required by seasonal conditions” by Orica Limited (ORI); by policies, e.g., “Working outdoors Excessive Heat Protocol, Severe Weather Management Plan, Severe Weather Action Plan” by APA Group (APA); air filters by Dexus Industria Reit (DXI); Heat risk policies by Mirvac Group (MGR), “Environment Health and Safety policy” by Sims Limited (SGM); by scheduling to avoid heat by Woodside Energy Group Ltd (WDS) and Fortescue metals Group Ltd (FMG); and by refurbishing buildings by Stockland (SGP). Quotes relating to health adaptation included:

*“An effective Health and Safety Management system is in place, and we adopt a risk-based approach to monitoring and managing the safety and wellbeing of our employees and contractors, this includes arrangements for exposure to extreme heat.”* in the report of TPG Telecom Limited (TPG).

and:

*“The health and wellbeing of our people is inherent in our culture and operational practices. For example, sun protection and hydration are regularly included as topics in site communications and safety briefings. Facilities in the North West of Australia experience high ambient temperatures. Major maintenance campaigns, where the number of people on site is significantly increased, are targeted for execution in the cooler months to minimise exposure to heat stress….”* in the report from WDS.

The climate report of Incitec Pivot Limited (IPL) explicitly links governance with the health impacts of climate change; “The [Committee] … assists the Board in overseeing … the management and governance of climate change issues relating to employee health and safety, such as heat stress and risks to our people associated with extreme weather events; emergency planning and response procedures for our operations relating to extreme weather events”. Other quotes relating to impacts, mitigation and adaptation are in Appendix Table A2.

In terms of health co-benefits of mitigation, one bank, ANZ Group Holdings Limited (ANZ), described lending products to encourage low carbon healthier homes. Three other reports mentioned improved air quality (AMP Limited (AMP), Downer EDI Limited (DOW), and Macquarie Group Limited (MQG)) but did not make a clear link with improved health although this might be assumed.

## 4 Discussion

Only 10.2 % of top ASX companies in 2023/24 had a stand-alone climate report but the existence of these reports is significant as it suggests that top companies acknowledged the need for separate climate change reporting. The finding that companies with climate reports had significantly greater market capitalisation may be due to greater resources as well as greater visibility and upcoming legislation being initiated with largest companies.

Health words were mentioned in almost all reports but the most common word across the 51 corporate climate reports was “emissions” which suggests that mitigation (i.e., reducing emissions) was the primary focus of these reports. Indeed, climate reports were more likely for companies in the Utilities sector than other sectors possibly reflecting self-awareness as high emitters. Environmental words typically occurred more frequently than health words which may be due in part to environmental legislation such as requirements for environmental assessments [40].

In this study of health and climate change engagement by companies, the Health Care sector had the lowest proportion of companies with climate reports perhaps indicating a lack of perceived relevance. This may be part of a general tendency in society for links not to be made between climate change and health.

Nevertheless, the content of the climate reports shows that companies appreciate they are vulnerable to and need to adapt to health and climate change impacts in complex ways, relating to their workers, customers and wider community. For example, one company observed that climate change would result in “potential reduced revenue due to lessened uptake of health insurance, supply chain delays and increased healthcare costs for members” (NHF). The health-related company, ORI, identified climate change reputational and transition risk due to changing societal expectations; “Societal standards for businesses to act responsibly are increasing. Failing to anticipate or respond could see increased regulatory burden, supply and/or operational disruption, damaged stakeholder relationships and reputation”. Reports point out that health issues could potentially influence climate change. For example, the pandemic could both increase and decrease climate change mitigation efforts: decrease mitigation due to perceived need for more single use plastic (Qantas Airways Limited (QAN)) and increase mitigation due to reduced emissions due to less aviation. Companies occasionally identified that they could provide adaptive solutions to the health impacts of climate change such as encouraging investment and building solutions.

In terms of potential drivers for a company to produce a corporate climate report, just over half (56.9 %) referred to the use of TCFD guidelines as their template and almost all (90 %) mentioned the TCFD. Multiple companies for these reports (which were identified in the study year of 2023) indicated that they were preparing for future mandatory Australian standards for climate disclosures. Health is not a key feature of the TCFD template although employee heat stress is touched upon. Inclusion of more references to health adaptation in mandatory guidelines could increase action on adaptation. Interestingly the Paris Agreement was clearly important for companies as it was mentioned in almost 85 % of reports.

In 2024, the Australian Commonwealth Corporations Act 2001 was amended to introduce climate-related financial disclosure requirements for large businesses and financial institutions in Australia [41]. These entities are now required to prepare a sustainability report that includes this information. The requirements commenced at the start of 2025, with the first reporting to be lodged early 2026. Entities are required to disclose information about climate-related risks to which the entity is exposed and climate-related opportunities available to the entity. Health is included as an aspect of climate-related physical risks. For example, the Australian Sustainability Reporting Standard for Climate-related Disclosures states that an entity's financial performance could be affected by extreme temperature changes impacting employee health and safety [42].

This study has limitations regarding the sensitivity and specificity of its methods. Underestimation of engagement in health and climate change may have occurred because the study focuses on standalone climate reports; hence such engagement in other reports such as the annual report and sustainability reports is not considered here.

Other limitations relate to the choice of health words which were based for consistency on those used by the Lancet Countdown’s climate change and health reports and may not capture all health-related references. On the other hand, the study did not specifically exclude non-human health words therefore there may be some overestimation of health content. This is likely to be the case with words such as vulnerable and stress which are used in many non-human contexts.

Finally, while this study analysed companies’ engagement with health and climate change through published climate reports, it does not measure companies’ implementation of actions and their success or otherwise. Measuring emissions over time may be one way to determine successful engagement but other measurable outcomes more clearly linked to reducing human health risk (such as by reducing morbidity) were not examined in this study.

## 5 Conclusion

Given the global scale and urgency of health issues related to climate change, much more engagement by all groups in society including companies is needed. This research finds that some of the top Australian companies produce a stand-alone climate report and most of these climate reports mention health impacts of climate change and adaptation to these health impacts. It is encouraging that these key companies have acknowledged the importance of climate issues. The adaptation issues discussed in the reports could be an example to other companies in the sector which are not already engaged. Future research could investigate whether health content of climate reports changes with mandatory reporting of climate change risk.

## CRediT authorship contribution statement

**Barbara P F Davis:** Writing – original draft, Methodology, Investigation, Data curation, Conceptualization. **Paul J Beggs:** Writing – review & editing, Validation, Supervision, Methodology, Conceptualization. **Petra L Graham:** Writing – review & editing, Validation, Supervision, Methodology, Formal analysis, Data curation.

## Declaration of competing interest

The authors declare no competing interests that could have influenced the work reported in this paper. There has been no financial support for this work that could have influenced its outcome.

## Appendix Supplementary materials

Image, application 1
