# Supplementary material for: Engagement with health in the climate reports of top corporations
Source: J Clim Chang Health. 2026 Jan 7;27:100643. doi: 10.1016/j.joclim.2025.100643 (PMC13184481; doi:10.1016/j.joclim.2025.100643)
Supplement: Supplementary file 1 [file mmc1.docx]

Appendix

## Table A1: Health words and other words from the 51 standalone corporate climate reports

| **Term** | **Description** | **Number (%) of reports with at least one mention** | **Median mentions (lower quartile, upper quartile)** | **Minimum, maximum mentions*** | **Total number of mentions** |
| --- | --- | --- | --- | --- | --- |
| Any health words |  | 49 (96.1) | 10.5 (6.0, 24.0), | 1, 82 | 888 |
| health | health | 38 (74.5) | 2.00 (0.75, 6.00) | 1, 26 | 177 |
| safety | safety | 35 (68.6) | 2.50 (0.00, 5.25) | 1, 22 | 189 |
| depression, anxiety, or stress | depression, anxiety, or stress | 32 (62.7) | 1.00 (0.00, 2.00) | 1, 32 | 113 |
| vulnerab* | vulnerable, vulnerability, etc. | 30 (58.8) | 1.00 (0.00, 2.00) | 1, 15 | 98 |
| homeless* or displace* | homeless, homelessness, displace, displaced, displacement | 21 (41.2) | 0.00 (0.00, 1.00) | 1, 4 | 37 |
| wellbeing or well-being | wellbeing or well-being | 21 (41.2) | 0.00 (0.00, 2.00) | 1, 16 | 54 |
| pandemic* | pandemic, pandemics | 15 (29.4) | 0.00 (0.00, 1.00) | 1, 9 | 39 |
| heat stress/ heat stroke | heat stress, heat stroke | 12 (23.5) | 0.00 (0.00, 0.00) | 1, 10 | 28 |
| air pollution or air quality | air pollution, air quality | 9 (17.6) | 0.00 (0.00, 0.00) | 1, 5 | 21 |
| healthcare or health care | healthcare, health care | 8 (15.7) | 0.00 (0.00, 0.00) | 1, 21 | 56 |
| disease* | disease, diseases, diseased, etc. | 7 (13.7) | 0.00 (0.00, 0.00) | 1, 3 | 9 |
| mobility | mobility | 7 (13.7) | 0.00 (0.00, 0.00) | 1, 8 | 20 |
| gender* | gender, genders | 6 (11.8) | 0.00 (0.00, 0.00) | 1, 3 | 9 |
| injur* | injury, injuries, injured | 6 (11.8) | 0.00 (0.00, 0.00) | 1, 6 | 11 |
| age, elderly or old people | age, elderly or old people | 4 (7.8) | 0.00 (0.00, 0.00) | 1, 1 | 4 |
| illness* | illness, illnesses | 4 (7.8) | 0.00 (0.00, 0.00) | 1, 2 | 19 |
| mental | mental | 3 (5.9) | 0.00 (0.00, 0.00) | 1, 2 | 4 |
| mortality or death | mortality, death | 3 (5.9) | 0.00 (0.00, 0.00) | 1, 1 | 3 |
| public health* | public health, etc. | 2 (3.9) | 0.00 (0.00, 0.00) | 1, 1 | 2 |
| vector* | vector, vectors | 2 (3.9) | 0.00 (0.00, 0.00) | 1, 1 | 2 |
| malaria* | malaria, malarial | 1 (2.0) | 0.00 (0.00, 0.00) | 1, 1 | 1 |
| mosquito* | mosquito, mosquitos | 1 (2.0) | 0.00 (0.00, 0.00) | 1, 1 | 1 |
| physical activit* | physical activity, physical activities | 1 (2.0) | 0.00 (0.00, 0.00) | 3, 3 | 3 |
| refuge* | refuge, refuges, etc. | 1 (2.0) | 0.00 (0.00, 0.00) | 2, 2 | 2 |
| ross river | Ross River Virus | 0 (0) |  |  |  |
| infectio* | infectious, infection, infections | 0 (0) |  |  |  |
| food poisoning, foodborne or food-borne | food poisoning, foodborne, food-borne | 0 (0) |  |  |  |
| stroke* | stoke, strokes | 0 (0) |  |  |  |
| morbidity | morbidity | 0 (0) |  |  |  |
| epidemic* | epidemic, epidemics | 0 (0) |  |  |  |
| occupational health and safety or ohs | occupational health and safety, ohs | 0 (0) |  |  |  |
| suicid* | suicide, suicides, suicidal | 0 (0) |  |  |  |
| obes* | obese, obesity | 0 (0) |  |  |  |
| dengue | dengue | 0 (0) |  |  |  |
| barmah forest | Barmah Forest Virus. Barmah Forest | 0 (0) |  |  |  |
| encephalitis | encephalitis | 0 (0) |  |  |  |
| diarrhoea* | diarrhoea, diarrhoeal | 0 (0) |  |  |  |
| malnourish*, malnutrition | malnourishment, malnourished, malnourish, malnutrition | 0 (0) |  |  |  |
| communicable disease* | communicable disease, communicable diseases | 0 (0) |  |  |  |
| sars | sars, SARS | 0 (0) |  |  |  |
| pneumonia* | pneumonia, pneumonias | 0 (0) |  |  |  |
| noncommunicable disease*, non-communicable disease*, ncd | noncommunicable disease, noncommunicable diseases, non-communicable disease, noncommunicable diseases, ncd, NCD | 0 (0) |  |  |  |
| measles | measles | 0 (0) |  |  |  |
| stunting | stunting | 0 (0) |  |  |  |
| epidemiolog* | epidemiological, epidemiology | 0 (0) |  |  |  |
| **OTHER WORDS** |  |  |  |  |  |
| economi*, economy, financial | Economic, economies, economy, financial | 51 (100) | 34.0 (20.5, 76.0) | 1, 238 | 2985 |
| environment*, biodiversity, flora and fauna, plants, animal*, nature | Environment*, environmental, biodiversity, flora and fauna, plants, animal, animals, nature | 51 (100) | 23.0 (11.5, 42.0) | 1, 376 | 2138 |
| customer*, communit*, client*, consumer*, shareholder*, investor* | customer, customers community, communities, client, clients, consumer, consumers, shareholder, shareholders, investor, investors | 49 (96.1) | 62.0 (20.0, 114.0) | 2, 742 | 4892 |
| employee*, worker*, staff, team* | Employee, employees, worker, workers, staff, team, teams | 49 (96.1) | 17.0 (7.5, 32.0) | 1, 133 | 1292 |
| legislat*, regulat*, law*, corporations act | Legislation, regulation, law, Corporations Act. | 48 (94.1) | 11 (6.0, 13.5) | 1, 130 | 1146 |
| tcfd, task force on climate-related financial disclosures | Task Force on Climate-Related Financial Disclosures | 46 (90.2) | 6.0 (3.0, 13.5) | 1, 47 | 465 |
| Paris Agreement | Paris Agreement | 43 (84.3) | 2.0 (1.0, 8.0) | 1, 22 | 277 |
| csr, corporate social responsibilit*, social licence, reputation | corporate social responsibility, CSR, responsibilities, social licence, reputation | 31 (60.8) | 2.0 (0.0, 1.5) | 1, 13 | 152 |
| cdp, carbon disclosure project | Carbon Disclosure Project | 19 (37.3) | 0.0 (0.0, 1.5) | 1, 191 | 734 |
| nger, national greenhouse and energy reporting | NGER, National Greenhouse and Energy Reporting | 16 (31.4) | 0.0 (0.0, 2.5) | 1, 16 | 117 |

*Among those reports with at least one mention

Table A2. Examples of health words by corporation and sector in 2023 in context. The full sentence in which the word appears is included.

| Category | Examples | Source | Page |
| --- | --- | --- | --- |
| Internal Impacts | Heat stress in particular can lead to reduced operational efficiency and worker injury. In extreme circumstances, it might lower our overall workforce supply. We can expect increasing labour costs due to greater downtime and health and safety compliance expenses. | SGP | 39 |
|  | The most material chronic physical risks are heat stress and drought with the former being pronounced across most parts of Australia and the latter being most pronounced in South Australia. | SGP | 46 |
|  | Worker health and safety  Description of impact, risks and opportunities.  Acute (extreme weather events) and chronic (increasing temperatures and number of hot days, increased prevalence of tropical diseases, etc.) physical climate change has the potential to increase health and safety risks for our employees and contractors, impacting productivity and absenteeism rates and our ability to attract and retain talent. | ORI | 43 |
|  | Flooding events can lead to operational disruptions and delays to projects due to unsafe working conditions and temporary site closures. Finally, stagnant water following floods may also lead to a rise in vector-borne diseases. | FMG | 31 |
| External Impacts | Rising mean temperatures  Increased risk of mosquitos and other exotic pests which pose a threat to New Zealand biodiversity and human health | AIA | 6 |
|  | The bushfires that impacted Australia in the 2020 summer were the worst in recorded history and Australian cities recorded the poorest air quality globally for approximately 30 days. The poor outdoor air quality presents challenges to maintaining healthy indoor environments for Dexus’s employees, tenants and customers across its managed portfolio. | DXI | 9 |
| External Mitigation | The cookstove project we are supporting, based in Kenya, helps to fight deforestation, reduces the amount of greenhouse gas emissions in the atmosphere, and improves people’s health. These stoves use 40-60% less wood and produce less smoke than ordinary ones. By investing in the local manufacturing and distribution of cleaner, cost-efficient household cookstoves, all projects contribute measurably to various SDGs and are Gold Standard certified. | WOR | 143 |
|  | ANZ Bank New Zealand Limited (ANZ Bank New Zealand) has seen growth in the number of New Zealand customers using business and retail lending products to reduce their carbon footprint and improve their home’s health and energy efficiency:  • Our Good Energy Home Loan top up is available to existing eligible1 home loan customers to upgrade their homes with solar panels, heating and insulation, double glazing, ventilation systems or rainwater tanks. It can also be used for electric and hybrid vehicles, electric bikes, and electric vehicle chargers. It allows customers to borrow up to NZ$80,000 at a 3-year fixed interest rate of 1 per cent per annum.  • Our Healthy Home Loan Package offers interest rate discounts and fee savings for eligible2 customers who are buying, building, renovating or already own a home with a  6 Homestar rating or higher.  Across the two products since October 2020,  8,091 households drew down the loans, for  an aggregate amount of NZ$372.7 million | ANZ | 19 |
| Internal Adaptation | The addition of humidity exacerbates heat stress for outdoor workers. Shift schedules may have to be adjusted to maintain safe working conditions and labour productivity may be impacted during heatwaves | FMG | 31 |
|  | Working Outdoors Excessive Heat Protocol, Severe Weather Management Plan, Severe Weather Action Plan | APA | 30 |
|  | An effective Health and Safety Management system is in place, and we adopt a risk-based approach to monitoring and managing the safety and wellbeing of our employees and contractors, this includes arrangements for exposure to extreme heat. | TPG | 9 |
|  | The Health, Safety, Environment and Community (HSEC) Committee of the Board assists the Board in overseeing the Group’s health, safety, environment and community (HSEC) performance and governance responsibilities, and the adequacy of the Group’s HSEC framework. This includes the management and governance of climate change issues relating to employee health and safety, such as heat stress and risks to our people associated with extreme weather events; emergency planning and response procedures for our operations relating to extreme weather events… | IPL | 7 |
|  | We can expect increasing labour costs due to greater downtime and health and safety compliance expenses. To manage these risks, we must integrate chronic physical risk assessments into our investment appraisal and development plans across all pipeline sites. | SGP | 39 |
|  | Under our SHES Group Standards, sites are required to monitor and maintain a safe working environment for employees. This includes monitoring workers and ensuring they are fit to work, and heat stress monitoring at our major operating facilities when required by seasonal conditions. | ORI | 43 |
|  | Refurbishing buildings to account for heat stress | SGP | 38 |
|  | Higher temperature and humidity, as well as an increasing  incidence of extreme heat events increases the risk of heat stress for our people at some of our sites. For this reason, we have incorporated the future climate scenarios developed for each of our 12 major manufacturing sites into Climate Change  Risk Review Packs to drive climate-related risk assessment throughout our risk management framework. The aim is twofold:  1. to ensure that the climate change-specific risks identified for each site during our most recent scenario risk analyses have been incorporated  into site risk registers and are being managed; and  2. to assist sites in identifying any existing operational risks which may be amplified by the expected changes in prevailing weather conditions at each site and ensure that any additional controls required are identified and assigned to risk control owners. | IPL | 23 |
|  | Our key goals as part of our Eraring people  transition strategy are to: ….providing health and wellbeing support, including superannuation and financial planning  and mental health training; | ORG | 30 |
|  | The health and wellbeing of our people is inherent in our culture and operational practices. For example, sun protection and hydration are regularly included as topics in site communications and safety briefings. Facilities in the North West of Australia experience high ambient temperatures. Major maintenance campaigns, where the number of people on site is significantly increased, are targeted for execution in the cooler months to minimise exposure to heat stress. Production planning and forecasting includes assumptions for ambient temperature, recognising that higher ambient temperatures can reduce plant performance. | WDS | 23 |
|  | Two of the key values in the Dexus Sustainability Approach are Future Enabled Customers and Strong Communities and Thriving People. They address direct material issues relating to the health, safety and security of Dexus’s employees and customers, including ensuring the IAQ of Dexus’s buildings are within the recommended PM10 range and that air filters in building HVAC systems are operating as expected. | DXI | 10 |
|  | Heat risk policies: Mirvac recognises the health and safety risks associated with hot days, particularly the higher risk of heat-related fatigue on construction sites. We have implemented policies specifically addressing heat risk and we continue to review and improve policies relating to  increases in temperature. | MGR | 13 |
|  | Sims Limited’s Environment Health and Safety (EHS) policy already covers heat stress. | SGM | 8 |
| External Adaptation | BlueScope has sponsored the Green Building Council of Australia to develop a new standard for sustainable Australian homes. The Standard, titled ‘Green Star Homes', will be used to assess Australian homes against three key criteria:  POSITIVE: net zero in energy, fully electric, draught sealed, efficient and powered by renewables.  HEALTHY: ventilated, comfortable, with products that are better for you.  RESILIENT: water efficient and climate change ready.  In addition to being net zero energy, Green Star Certified homes will need to be built with proactive measures to be better than Code at withstanding natural disasters and future climate change impacts such as bushfires, flooding, and heat stress. | BSL | 60 |
